# CRISPR-Cas9 approach confirms Calcineurin-responsive zinc finger 1 (Crz1) transcription factor as a promising therapeutic target in echinocandin-resistant *Candida glabrata*

**DOI:** 10.1371/journal.pone.0265777

**Published:** 2022-03-18

**Authors:** Andres Ceballos-Garzon, Elvira Roman, Jesús Pla, Fabrice Pagniez, Daniela Amado, Carlos J. Alméciga-Díaz, Patrice Le Pape, Claudia M. Parra-Giraldo

**Affiliations:** 1 Unidad de Proteómica y Micosis Humanas, Grupo de Enfermedades Infecciosas, Departamento de Microbiología, Facultad de Ciencias, Pontificia Universidad Javeriana, Bogotá, D.C., Colombia; 2 Department of Parasitology and Medical Mycology, Faculty of Pharmacy, University of Nantes, Nantes, France; 3 Departamento de Microbiología y Parasitología, Facultad de Farmacia, Instituto Ramón y Cajal de Investigaciones Sanitarias (IRYCIS), Universidad Complutense de Madrid, Madrid, Spain; 4 Institute for the Study of Inborn Errors of Metabolism, Faculty of Science, Pontificia Universidad Javeriana, Bogotá, D.C., Colombia; University of California Riverside, UNITED STATES

## Abstract

Invasive fungal infections, which kill more than 1.6 million patients each year worldwide, are difficult to treat due to the limited number of antifungal drugs (azoles, echinocandins, and polyenes) and the emergence of antifungal resistance. The transcription factor Crz1, a key regulator of cellular stress responses and virulence, is an attractive therapeutic target because this protein is absent in human cells. Here, we used a CRISPR-Cas9 approach to generate isogenic *crz1*Δ strains in two clinical isolates of caspofungin-resistant *C*. *glabrata* to analyze the role of this transcription factor in susceptibility to echinocandins, stress tolerance, biofilm formation, and pathogenicity in both non-vertebrate (*Galleria mellonella*) and vertebrate (mice) models of candidiasis. In these clinical isolates, *CRZ1* disruption restores the susceptibility to echinocandins in both *in vitro* and *in vivo* models, and affects their oxidative stress response, biofilm formation, cell size, and pathogenicity. These results strongly suggest that Crz1 inhibitors may play an important role in the development of novel therapeutic agents against fungal infections considering the emergence of antifungal resistance and the low number of available antifungal drugs.

## Introduction

In the last years invasive fungal infections (IFI), which are associated with around 1.6 million deaths (similar to tuberculosis and 3-fold higher than malaria infections) have become a public health problem [1, 2]. *Candida*, *Aspergillus*, *Cryptococcus*, and *Pneumocystis* are the main fungal pathogens responsible for the majority cases of serious fungal diseases. Among the genus *Candida*, *Candida albicans* is the most commonly isolated species, with close to 30% mortality rates in candidemia [3, 4]. However, non*-C*. *albicans* species such as *Candida glabrata* have emerged as a frequent cause of life threating IFI becoming the second or third most frequent species. For this species, the associated mortality is about 50% [5, 6].

While current therapeutic options for IFI are limited to only three classes of drugs (polyenes, azoles and echinocandins), the emergence of resistant strains to some of these molecules is even more concerning. In the case of *C*. *glabrata*, antifungal resistance mechanisms have been relatively well characterized [7, 8]. It is known that this species, commonly resistant to azoles, has high genetic plasticity and can acquire resistance to other drugs easily [8, 9]. *C*. *glabrata* shows increasing resistance to echinocandins, which is now considered the first-line of therapy for candidemia [10]. The main resistance mechanism is associated with mutations in the *FKS1* and/or *FKS2* genes that encode the subunits of the 1,3-β-D-glucan synthase protein, the target of echinocandins, leading to multi-drug resistance (MDR) [7, 11].

Although there is an urgent need to develop new antifungal drugs, the close sequence homology of targets between the host and the pathogen makes this task challenging [12, 13]. However, in recent years, some metabolic pathways have become attractive targets due to their role in host adaptation and cellular stress responses [14]. Among these, signaling pathways have been broad studied such as the Ras/cAMP/PKA pathway, calmodulin/calcineurin pathway (CaM/CaL), TOR (target of rapamycin) and mitogen-activated protein kinase (MAPK) signaling pathways [15–18].

The calmodulin/calcineurin (CaM/CaL) is a conserved pathway from fungi to humans [19]. In yeast, this pathway is involved in ion homeostasis, sphingolipid and cell wall biosynthesis, protein trafficking, ubiquitin signaling, autophagy, adaptation to stress and most importantly, in antifungal resistance [20–23]. A key element of this route is the complex formed by the proteins Cnb1, Cna1, Hsp90 and the transcription factors Crz1 (calcineurin-responsive zinc finger 1) in yeast or the NFAT (nuclear factor of activated T cells) in mammals. Upon activation by dephosphorylation (a process mediated by the CaM/CaL-Hsp90 complex), Crz1 translocates to nucleus and through its C_2_H_2_ zinc finger motif binds to a specific element in the CDRE gene promoter (calcineurin dependent response element), initiating the activation of about 87 genes, among which is the *FKS2* gene [24–27]. *C*. *albicans and C*. *glabrata* lacking CaM/CaL fail to survive in the presence of membrane stressors, as occurs in *S*. *cerevisiae* mutants, have a reduced MIC to antifungals and have attenuated virulence in mouse models [28, 29]. In *C*. *glabrata* and *C*. *lusitaniae*, calcineurin-Crz1 signaling controls the virulence in a murine model of systemic infection [26, 30]. Growth defects at alkaline pH and at elevated temperature have also been reported in different *crz*1Δ *Candida* species [31, 32]. Nevertheless, the implication of Crz1 in stress response, biofilm formation and cell morphology in echinocandin-resistant *C*. *glabrata* clinical isolates has not been evaluated. Furthermore, susceptibility of *Crz1*Δ mutants of these resistant isolates has also to be evaluated for a better understanding of the putative therapeutic impact of this target.

Over the last decade, the innovative discovery of clustered regularly interspaced short palindromic repeats (CRISPR)-Cas9 has revolutionized genome editing in many organisms, including fungi [33, 34]. However, few studies have been published using CRISPR-Cas9 in *C*. *glabrata* [35–38]. This approach can be used to transform and accelerate drug discovery and development by enabling a fast and accurate editing of genomic information in biological model systems [39, 40]. This study aimed to assess the role of Crz1 in the resistance to caspofungin in two *C*. *glabrata* resistant clinical isolates. For this purpose, we used a CRISPR-Cas9 genome editing approach to generate isogenic *crz1*Δ strains in clinical isolates and analyzed the role of this transcription factor in the resistance to echinocandins, tolerance to stress conditions, biofilm formation and cell wall composition. We have also addressed the virulence of the obtained mutants in a non-vertebrate (*Galleria mellonella*) and vertebrate (Swiss mouse) models of systemic candidiasis, supporting the role of this factor as a promising antifungal target in this clinically relevant yeast.

## Materials and methods

### Isolates and media

Two echinocandin-resistant isolates CAGL1875 and CAGL1256 obtained from blood and urine cultures of hospitalized patients in intensive care unit of Centre Hospitalier Universitaire de Nantes, France were described in a previous study by our research group [20, 41]. Isolates were streaked from a glycerol stock onto yeast extract peptone dextrose agar (YPD) and grown for 24 h at 37 °C. Genome sequencing is available in the NCBI BioProject database with the accession number PRJNA692260.

### Construction of CRISPR-Cas9 mutants for CgCRZ1

*C*. *glabrata* isolates CAGL1875 and CAGL1256 were used as the parent strain to construct the CRISPR-Cas9 mutants for *CgCRZ1*. sgRNAs were generated by phosphorylation and annealing of complementary single stranded DNA oligonucleotides sg*CRZ1*-up (GATCGATAACCATAATTTCTCGACCG) and sg*CRZ1*-rev (AAAACGGTCGAGAAATTATGGTTATC) and inserted into the pV1083 vector previously digested with *BsmB* I and treated with calf intestinal phosphatase CIP (NEB) to generate the recombinant plasmid pV1382-sgRNA. The insertion of the guide was confirmed by sequencing with the o-seq-sgRNA primer (GGCTAGCGGTAAAGGTGCG). Repair templates (Mutagenic template-up: GCATGAATGGCGATGTATATGAACAAGATAACAATAACCATAATTTCTCGACCTAATGAGGATCCCAGAATAT and Mutagenic template-rev: TGAAAATGTAGACTCACTCAGTATATTCTGGGATCCTCATTAGGTCGAGAAAT) were generated with 53-bp oligonucleotide primers containing 23-bp overlaps at their 3´ ends centred at the mutation point, which consisted of two stop codons. Primers *CRZ1*-F (GCATGAATGGCGATGTATATGAAC) and *CRZ1*-R (TGAAAATGTAGACTCACTCAGTATATTCTG) were used to amplify the repair donor DNA fragment. The donor DNA and the recombinant plasmid pV1382-SgRNA were used to transform *C*. *glabrata* cells CAGL1875 and CAGL1256 by electroporation [42].

Transformed yeasts were growth in YPD supplemented with nourseothricin selection marker of potentially CRISPR-Cas9 edited mutants. Nourseothricin-resistant clones were evaluated by detection with PCR and *BamHI*-digestion analysis. PCR was performed on genomic DNA using the primers *CRZ1*-CF (GGGTTTTCTGTCAAAACCACTGC) and *CRZ1*-CR (CAAATGGATCTCCTCCAATTAATTGC). Finally, *CRZ1* mutants were confirmed by sequencing.

### Antifungal susceptibility testing

Antifungal susceptibility testing was conducted using the Clinical and Laboratory Standards Institute broth microdilution method (CLSI-BMD), following the M27-A3 guidelines [43]. Briefly, Isolates and mutants were streaked from a glycerol stock onto YPD and grown for 24 h at 37 °C. Colonies were suspended in 1 mL phosphate buffered saline (PBS) and diluted in liquid RPMI 1640 medium to 10^3^ cells/mL in a 96-well plate, containing a gradient of two-fold dilutions per step of antifungal (CAS, MCF), with the first well contain no drug. MICs were visually, and densitometry determined as the lowest concentration of drug that caused a significant diminution (MIC/2 or ≥50%) compared with that of the drug-free growth control after 24 h of incubation. Quality control was ensured by testing the CLSI-recommended strains *C*. *parapsilosis* ATCC 22019 and *C*. *krusei* ATCC 6258 [44].

### Stress-related phenotypic assays

To examine the potential role of Cg*CRZ1* mutant’s cellular protection to heat and oxidative stresses, associated or not to caspofungin was assessed. For heat-shock stress, drop tests were performed by spotting serial dilutions of *C*. *glabrata* (10^6^ to 10^3^cells/mL) onto YPD agar plates with CaM/CaL inhibitors fluphenazine (Fph), tacrolimus (Fk506), cyclosporin A (CsA) (15 μg/mL), caspofungin (1 μg/mL) or both compounds. The plates were incubated at 37°C and 40°C for 24 h. For oxidative stress, YPD plates were prepared as previously except that the medium was supplemented with the naphthoquinone menadione (0.2 and 0.4 mM), a cytotoxic quinone that generates superoxide. The plates were incubated at 37°C for 24 h [20].

### Confocal microscopy

Yeast cells collected by centrifugation (4000 g, 5 min,) and suspended in 200 μL phosphate buffered saline were stained with 10 μg/mL concanavalin A–Alexa Fluor 594 conjugate (Molecular Probes, Eugene, OR) and 20 μg/mL Fluorescent Brightener 28 (Sigma) for 25 min in the dark. Then, cells were washed twice in PBS and fixed with 4% paraformaldehyde for 30 min. Fluorescence-stained sections were examined under a Nikon A1 RSI microscope with a magnification of ×60 at constant Z-steps of 1 μm. The laser confocal system comprised a 65-mW multi-Ar laser. Three-dimensional (3D) images were processed with NIS elements version 3.21 (Nikon Instruments Inc.) and Volocity 3D image analysis software version 6.01 (PerkinElmer). Cell size measurements and total cell volume were determined using ImageJ software.

### Biofilm formation

*C*. *glabrata* isolates and mutants were grown in Sabouraud medium (Biomerieux, France) and incubated at 37°C for 24 h. Two hundred μL of *Candida* cell suspensions (10^6^ cells/mL) in RPMI-1640 (SIGMA^®^, Saint Quentin Fallavier, France) with MOPS adjusted to pH 7 were seeded in 96-well microdilution wells with GDHK-1325 250mm Gam polyurethane catheter pieces (Hechingen, Germany) and allowed to adhere for 24 h at 37°C. The non-adherent cells were removed by redisposing catheter pieces in new microplates wells. Follow by incubation for 24 h at 37°C for biofilm formation phase. Then, catheter pieces were washed twice with PBS and finally 100 μL of RPMI-1640 plus 10 μL of resazurin (700μM) was added to each well and incubated at 37°C for 2–4 h [45]. Finally, fluorescence was measured at 560 nm with an emission at 590 nm. The results were expressed in arbitrary fluorescence unit (AU). Statistical analysis was performed using Graph Pad Prism version 5.0, Software Inc., La Jolla, CA, USA.

### Invertebrate *Galleria mellonella* model

Killing assays were performed in *G*. *mellonella* as described by Fallon, 2012 [46]. Briefly, larvae of late stages (fifth and sixth) between 250 to 330 mg and a length of approximately 2 cm were selected. A group of 10 larvae was used for the following controls: absolute control, disinfection, and inoculation. To compare mortality three biological replicates were performed with 10 larvae for each isolate evaluated. *C*. *glabrata* parent and mutant isolates were grown in Sabouraud dextrose agar and incubated for 48 h at 37°C. Suspensions adjusted to 1x10^9^ UFC/mL using Neubauer chamber were used to inoculate 10 larvae per *Candida* isolate. Larvae receive 10 μL of inoculum and 10 μL of caspofungin (100 mg/L), by injection into the last left and right pro-leg respectively using a 0.5mL (BD^®^) gauge insulin syringe. After inoculation, larvae were placed in Petri dishes and incubated in darkness at 37°C. The larvae were monitored for 10 days, and survival outcome was determined; larvae were considered dead when no response was observed following touch.

### Mice use and care

Females Swiss mice (Janvier Labs, Le Genest-Saint-Isle, France) with a body weight of ~29 g were obtained and allowed to acclimate for 7 days prior to use. Environmental controls for the animal room were set to maintain a temperature of 16 to 22°C, a relative humidity of 30 to 70%, and a 12:12 hourly light-dark cycle. All efforts were made to minimize suffering.

According to humane endpoints defined by the Animal Welfare Organization of the animal house, a score is calculated as a function of observations made. Based on this score and on the thresholds reached, corrective measures have been defined to limit the pain and suffering of animals ranging from improvement of well-being to euthanasia.

After inoculation of the Candida suspension, the animals were observed twice a day and scored daily. It consists of evaluating and assigning a score ranging from 0 to 2 for each of the following parameters: temperature, appearance of the hair, general morphology, weight, physical appearance of the face (eyelids, eye, vibrations), behavioural appearance (group behaviour, activity, motor deficit, spasms/tremors) and breathing.

A score between 8 and 14 corresponds to moderate pain and involves the use of opioid analgesic: buprenorphine. It is used at the dosage of 0.05 mg/kg in SC, 2 to 3 times a day, depending on the evolution of the state of the mouse. Experience shows that the duration of administration is short (<24h), the evolution of systemic candidiasis being sudden and rapid. The worsening of the condition is of very poor prognosis. Thus, animals with a score above 15 are classified as seriously ill and euthanized.

### Systemic *C*. *glabrata* infection model

Mice were immunosuppressed by subcutaneous injection of 30 mg/kg prednisolone (Hydrocortancyl^®^) one day before challenge. On day 0, mice were infected intravenously (100 μL) with a blastoconidia suspension (10^8^ yeast/mL) of WT CAGL1875 clinical isolate (*in vitro* caspofungin-resistant, 19 mice) or the *crz1*Δ *C*. *glabrata* (*in vitro* caspofungin-susceptible, 20 mice). One hour after infection, mice of each group (WT and *crz1*Δ) were separated in 2 sub-groups that were treated intraperitoneally once daily for 4 days. One received a 2.5 mg/kg body weight of caspofungin (OHRE Pharma, Tours, France) treatment, the other (control sub-group) received 100 μL of distillate water. Survival was monitored for 7 days post inoculation. No mice died during the experiment.

At the end of the assay (day 7), the spleen and kidney of euthanized mice (by cervical dislocation) were excised and weighed. Tissues were homogenized and serially diluted 100 to 10000-fold in sterile saline solution, then plated onto Sabouraud dextrose agar and incubated for 48 h to determine the number of colony-forming units (CFUs). Tissue fungal burden was expressed as the average Log(CFU)/gram of tissue. Mean CFUs in kidneys and spleens were compared between WT and *crz1*Δ *C*. *glabrata* and with vehicle control.

### Ethical approval

The mice experimental protocol was approved by the Ethic Committee on Animal Testing (committee N°006) and was authorized by the French Ministry of Higher Education, Research and Innovation (APAFIS≠9710–2017042410186554 v4).

### Statistical analysis

Experiments were performed on three independent biological replicates; survival curves were constructed using the method of Kaplan and Meier, then the curves were compared using the Log-Rank (Mantel-Cox) test. For the mouse model, statistical comparisons were made by analysis of variance (Two-way ANOVA) followed by a Tukey–Kramer post hoc test. Statistical models were constructed and analyzed using GraphPad Prism version 8.0 (GraphPad Software, San Diego, CA, USA). A p-value <0.05 was considered statistically significant. Cell size measurements were done using the following formula: 4/3*π*[r3(diameter: width + height / 2 = average diameter/2 = radius)].

## Results

### Disruption of *CRZ1* in clinical isolates using CRISPR-Cas9

**Fig 1:**
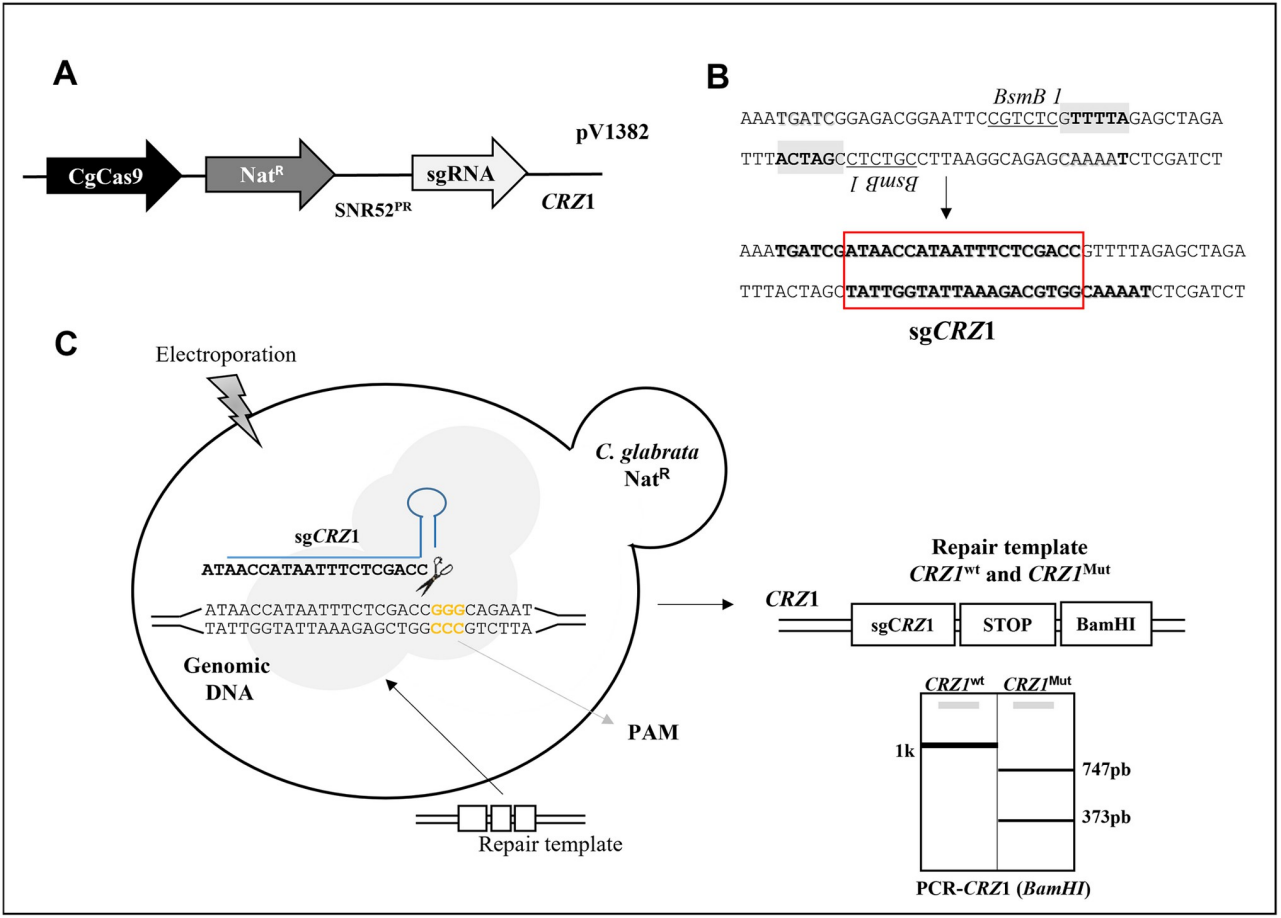
Construction of CRISPR mutants for *CgCRZ*1. **A**. The plasmid pV1382 contains CgCAS9, the nourseothricin resistant gene (Nat^R^) and *SNR52* promoter that control the expression of the desired sgRNA. **B**. pV1382 permits a rapid cloning by *BsmB* I digestion followed by ligation of annealed oligos (boldface type) containing the Cg*CRZ1*sequence of synthetic guide RNA (in red bracket). **C**. Scheme of Cg*CRZ1* mutagenesis mediated by CRISPR/Cas9. sg*CRZ1* guides the nuclease Cas9 to the specific genomic site and allows DSB next to protospacer adjacent motif (PAM). Homology directed repair (HDR) with a repair template containing two stop codons (TAA-TGA) and one *BamH*I restriction site allows further analysis of *crz1* mutants by PCR.

*crz1Δ* mutants were generated using the new unified solo vectors developed by Vyas *et al*., that allows efficient genetic engineering in *C*. *glabrata* incorporating both Cas9 and sgRNA into a single vector with a dominant marker [38]. *In silico* analysis of the *CRZ1* orf using the Chop-Chop server (https://chopchop.cbu.uib.no) was used to identify sequences likely to be used as CRISPR guides. A guide starting at 215 bp from the starting methionine was selected and cloned into the expressing vector. This plasmid was used to transform the CAGL1875 and CAGL1256 clinical isolates, which have a caspofungin-resistant phenotype with a repair template which incorporate double-stop codon and a *BamHI* restriction site. Nourseothricin-resistant colonies were then checked for the correct *CRZ1* disruption by *CRZ1* amplification followed by *BamHI* digestion. Finally, the clones that showed a double band in agarose gel were confirmed by sequencing (Fig 1).

### *CRZ*1 disruption restores caspofungin-susceptibility phenotype in resistant *C*. *glabrata* clinical isolates

**Fig 2:**
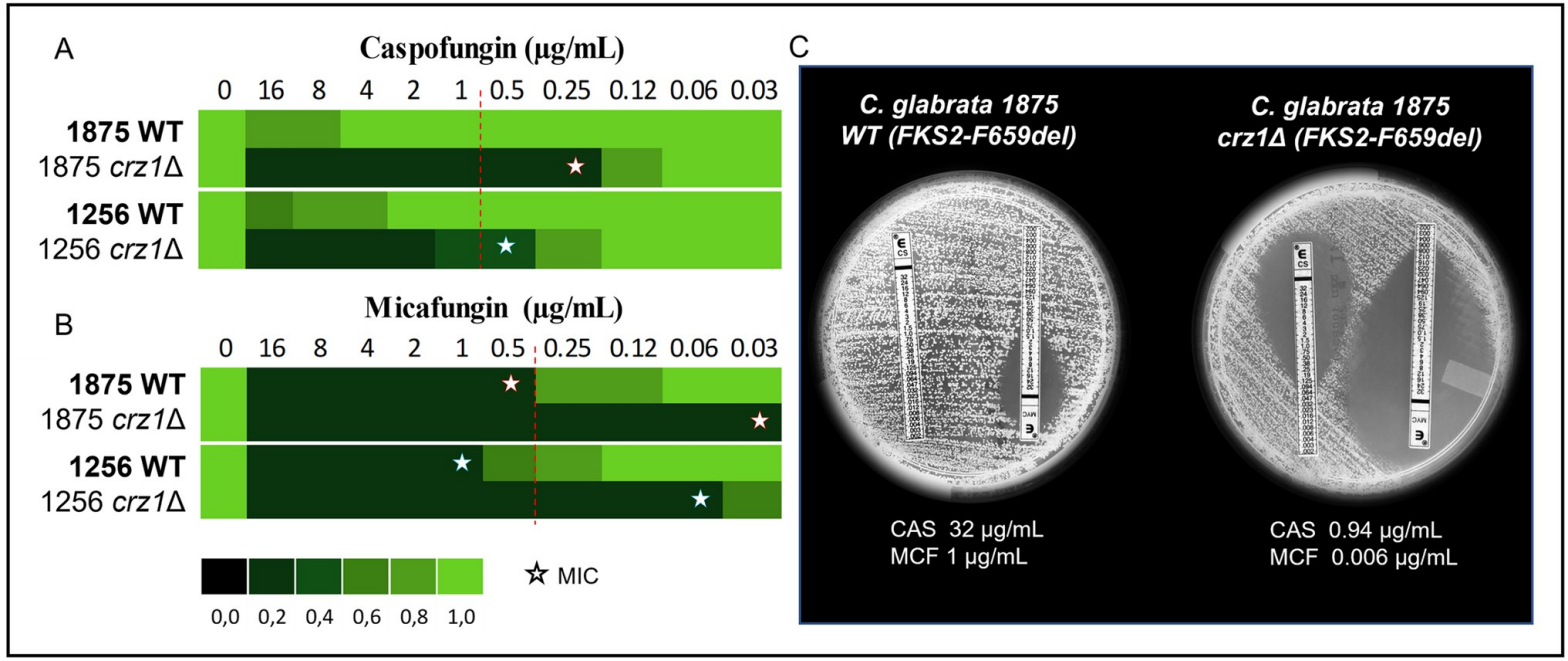
Susceptibility determination. **(A)** Caspofungin **(B)** Micafungin, minimal inhibitory concentration (star) by broth microdilution method. The green colour bar represents relative growth. Dotted red line indicates cut-off values **(C)** Caspofungin (CAS) and micafungin (MCF) Etest determination using RPMI agar supplemented with 2% glucose. The 10^6^ cell/mL yeast suspensions were spread on RPMI agar plates, MIC readings were made following 24 h incubation at 35 °C. The MIC was read as the drug concentration that leads to 80% of inhibition.

The *C*. *glabrata* isolates CAGL1875 and CAGL1256 which exhibited a deletion in the *FKS*2 gene (F659del) were previously reported as resistant to caspofungin (MIC >16μg/mL) by our group [20]. Micafungin resistance was confirmed with MIC values (> 0.5μg/mL). The disruption of *CRZ1* allowed the restoration of echinocandin-susceptibility of the two isolates, since the *crz1*Δ mutants showed low MIC values for caspofungin (MIC 0.25 and 0.5 μg/mL) and micafungin (MIC 0.03 and 0.06 μg/mL), respectively (Fig 2).

### *CRZ1* disruption does not affect thermotolerance but leads to increased susceptibility to oxidative stress

**Fig 3:**
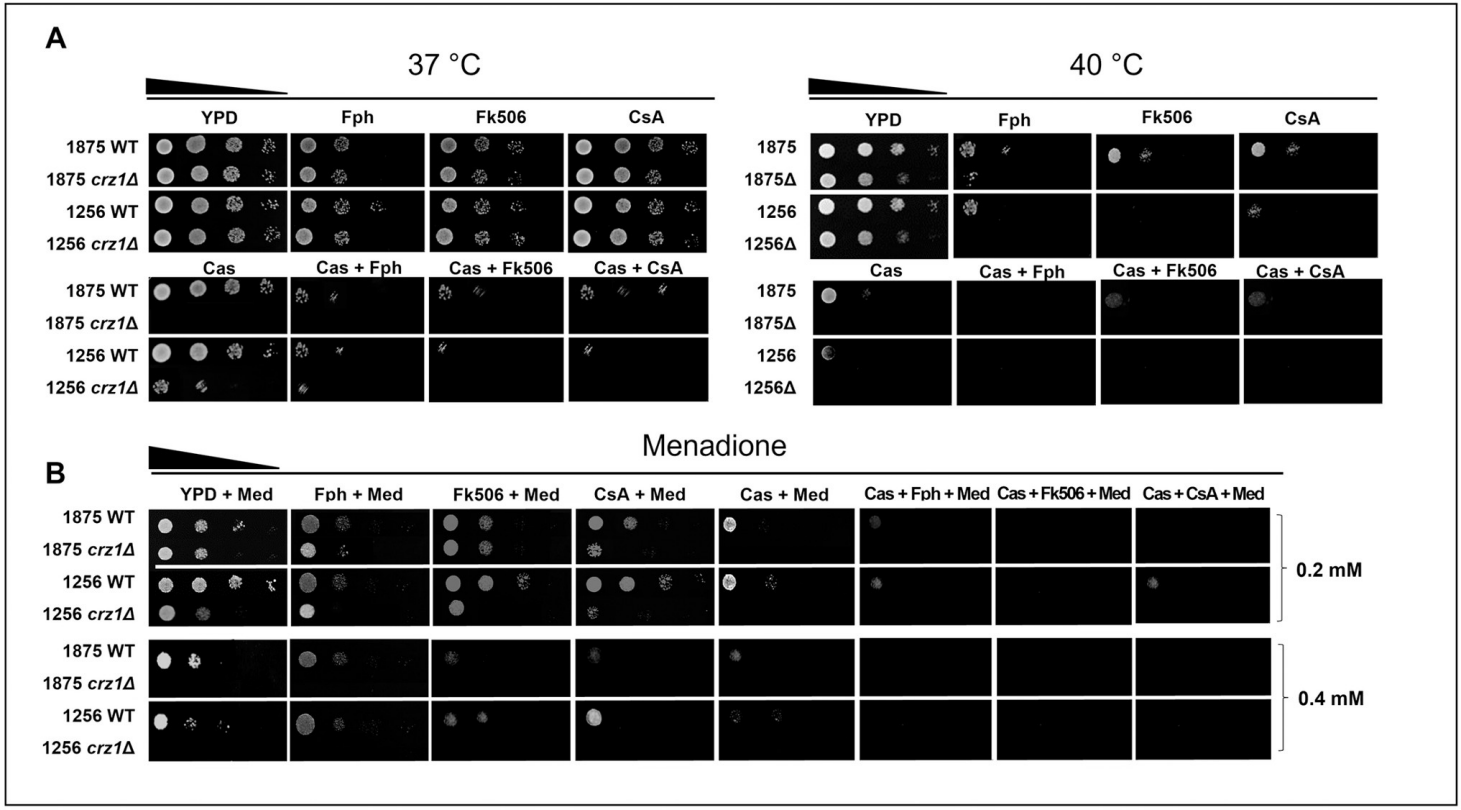
Stress responses. **(A)** Isolates were heat-shocked at 37 and 40°C with 15 μg/mL CaM/CaL inhibitors (fluphenazine (Fph), tacrolimus (Fk506), cyclosporin A (CsA)) and with or without 1 μg/mL caspofungin (Cas) **(B)** and were tested for oxidative stress with 0.2 and 0.4 mM menadione with or without CaM/CaL inhibitors.

To understand the heat and oxidative stress response in *crz1Δ* mutants, we analyzed growth in the presence of menadione and compared growth at 37 and 40 °C in solid drop assays. Spot test at 37 °C or after heat shock at 40 °C did not show major differences between the growth of mutants and WT isolates. However, at 37°C the use of CaM/CaL inhibitors (Fph, Fk506 and CsA) slightly affected the growth, and the association of the CaM/CaL inhibitors with caspofungin seriously compromised it, being more pronounced for the mutants. At 40 °C, the presence of CaM/CaL inhibitors alone and in combination with caspofungin strongly affected the growth (Fig 3A). Regarding oxidative stress, the addition of 0.2 and 0.4 mM menadione caused a significant decreased growth of mutants compared with their parental isolates. Moreover, *crz1Δ* mutants were mostly affected in the presence of CaM/CaL inhibitors and unable to grow in presence of caspofungin. At 0.4 mM menadione, the WT isolates showed a slight reduction of growth in presence of calcineurin inhibitors whereas the *crz1Δ* mutants were unable to grow at this concentration. The combination with caspofungin completely inhibited growth of all the strains (Fig 3B).

### *crz1*Δ mutants display changes in cell wall composition and yeast size

**Fig 4:**
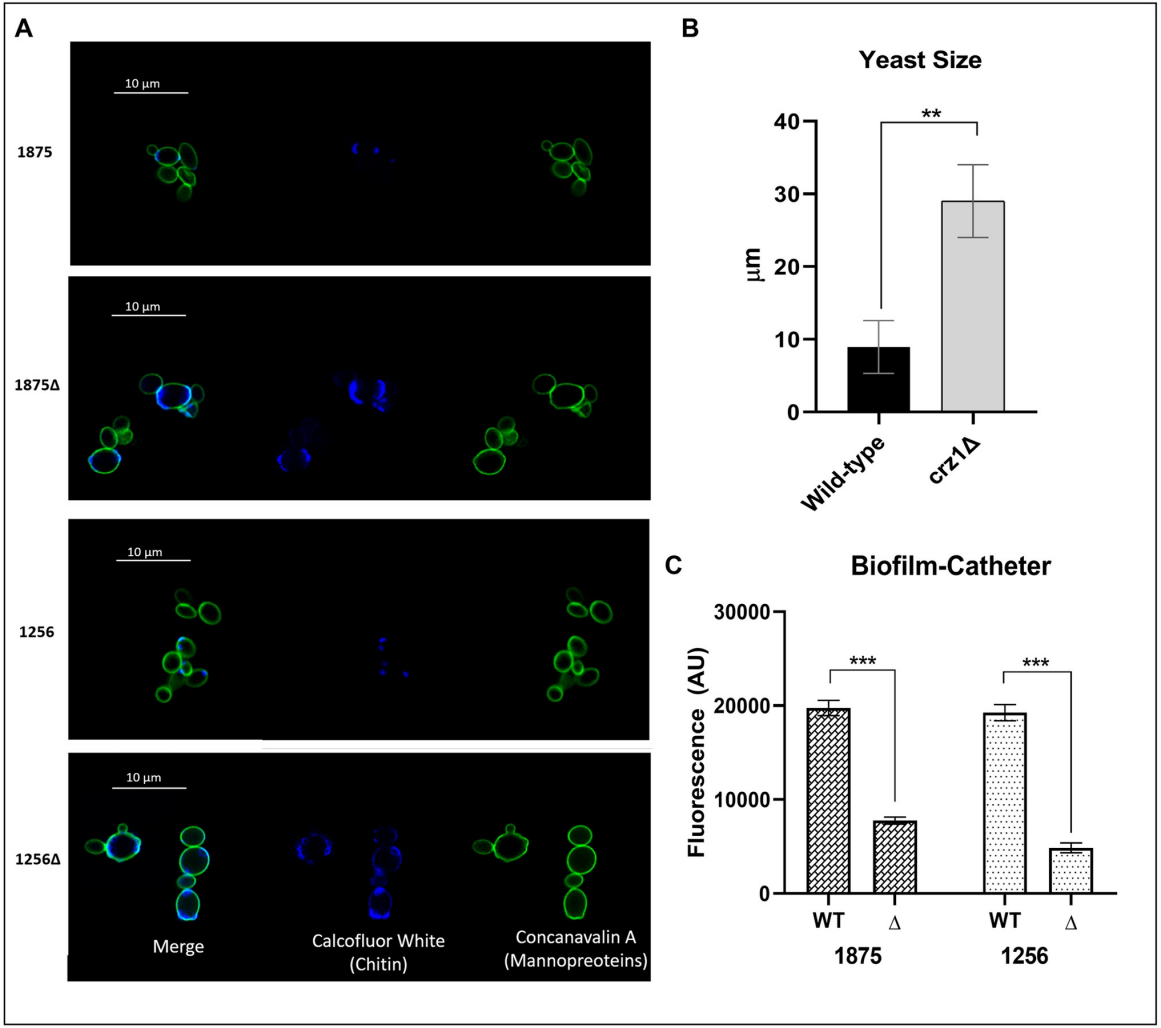
Confocal images and biofilm formation of *C*. *glabrata* isolates. **A** Yeast were stained with calcofluor white and concanavalin A conjugate for confocal scanning laser microscopy visualization. Representative images were shown. **B** Measurement of yeast cell size. **C** Biofilm formation on catheter pieces. Data is expressed as arbitrary fluorescence units. P-values of < 0.05 were used to indicate statistical significance. As follows, p < 0.05* p < 0.02** and p < 0.001***.

Different responses to caspofungin could be caused by differences in the cell wall among strains. We therefore analysed cell wall composition of the indicated strains by staining cells with concanavalin A (ConA), a lectin probe with strong affinity for yeast α-mannans and calcofluor white, which binds chitin. We observed that ConA staining was similar in both the mutants and parental strains and the fluorescent green colour was homogeneously distributed throughout the cell wall of all the strains. By contrast, staining with calcofluor white showed clear differences between mutants compared with their parental isolates, with a few preferences toward bud scars (Fig 4A). Interestingly, we observed that *CRZ1* disruption in WT clinical isolates resulted in increased cell sizes, reaching values of 29 ± 4 μm compared to (9 ± 3 μm of the WT strains (*p* < 0.02) (Fig 4B).

### *crz1*Δ mutants exhibit a reduced capacity of biofilm formation

To evaluate the role of Crz1 in biofilm formation, we compared the ability to form biofilms using the polyurethane catheter pieces methodology of *crz*1Δ mutants and their parental isolates. Both clinical isolates were able to form biofilm, but biofilm development was significantly reduced when *CRZ1* was disrupted (threefold decrease *p* <0.001) (Fig 4C).

### Disruption of *CRZ*1 results in a decreased virulence in two different animal models

**Fig 5:**
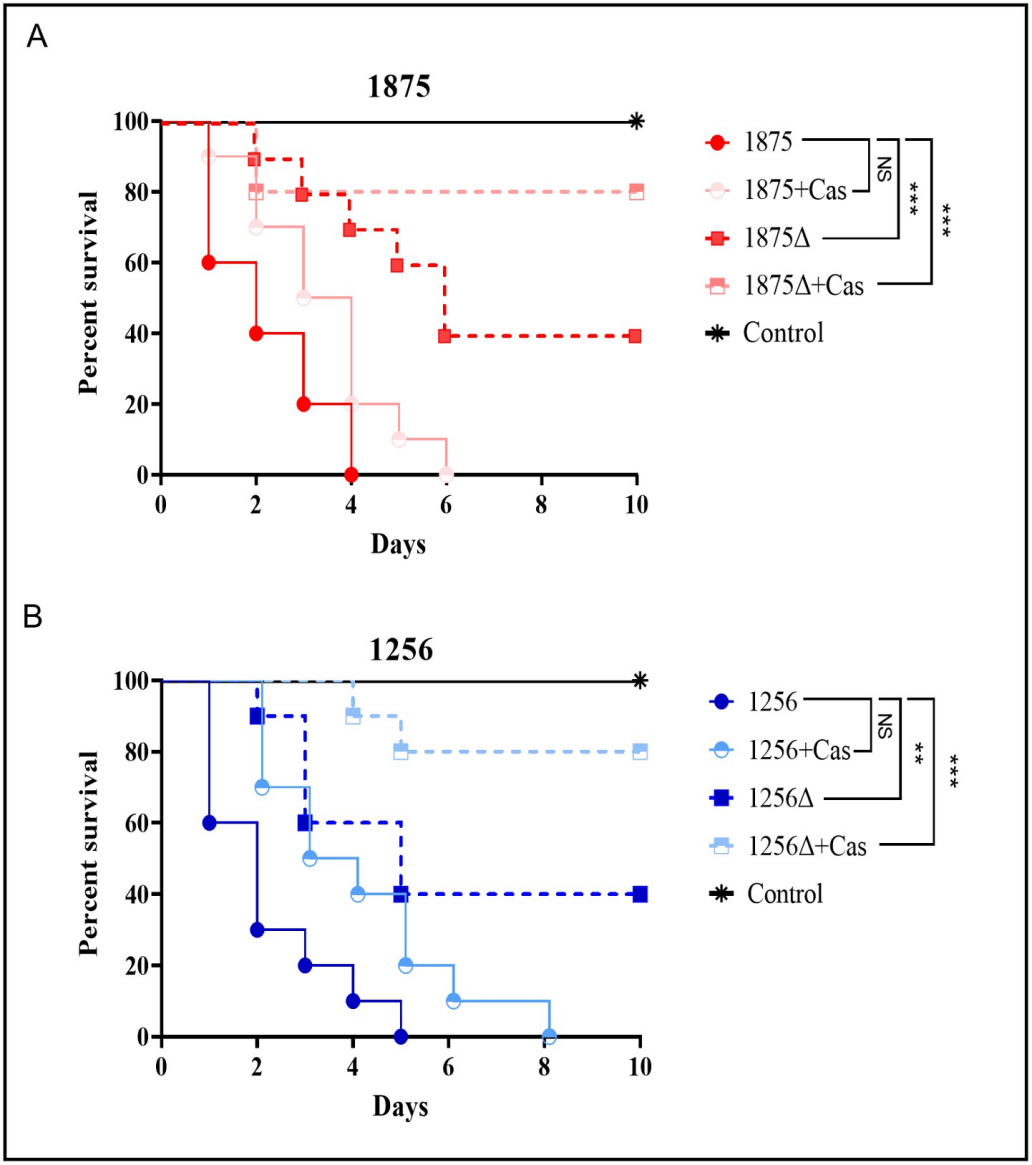
Time-kill curves. **A** *C*. *glabrata* 1875 **B** 1256, wild type (circle) and *crz1Δ* (square) exposed to caspofungin 100 mg/L. The data are expressed as the percentages of survival. Log-rank (Mantel–Cox) test with p-values of < 0.05 was used to indicate statistical significance. As follows, p < 0.05* p < 0.02** and p < 0.001***. Not significant (NS).

Virulence was first addressed in the non-vertebrate model of *Galleria mellonella*. *C*. *glabrata crz1*Δ mutants exhibited a significantly attenuated virulence compared with the WT isolates (*p* < 0.005). Indeed *C*. *glabrata* WT CAGL1875 and CAGL1256 caused 100% mortality of larvae by day 4 and 5, respectively, whereas *crz1*Δ mutants caused 60% mortality after 10 days of follow-up. In contrast to what occurred for WT isolates, treatment with caspofungin at 1 μg/larvae proved to be effective in prolonging survival of larvae infected with *crz1*Δ mutants, which exhibited 80% of survival at day 10 of experiment (Fig 5).

**Fig 6:**
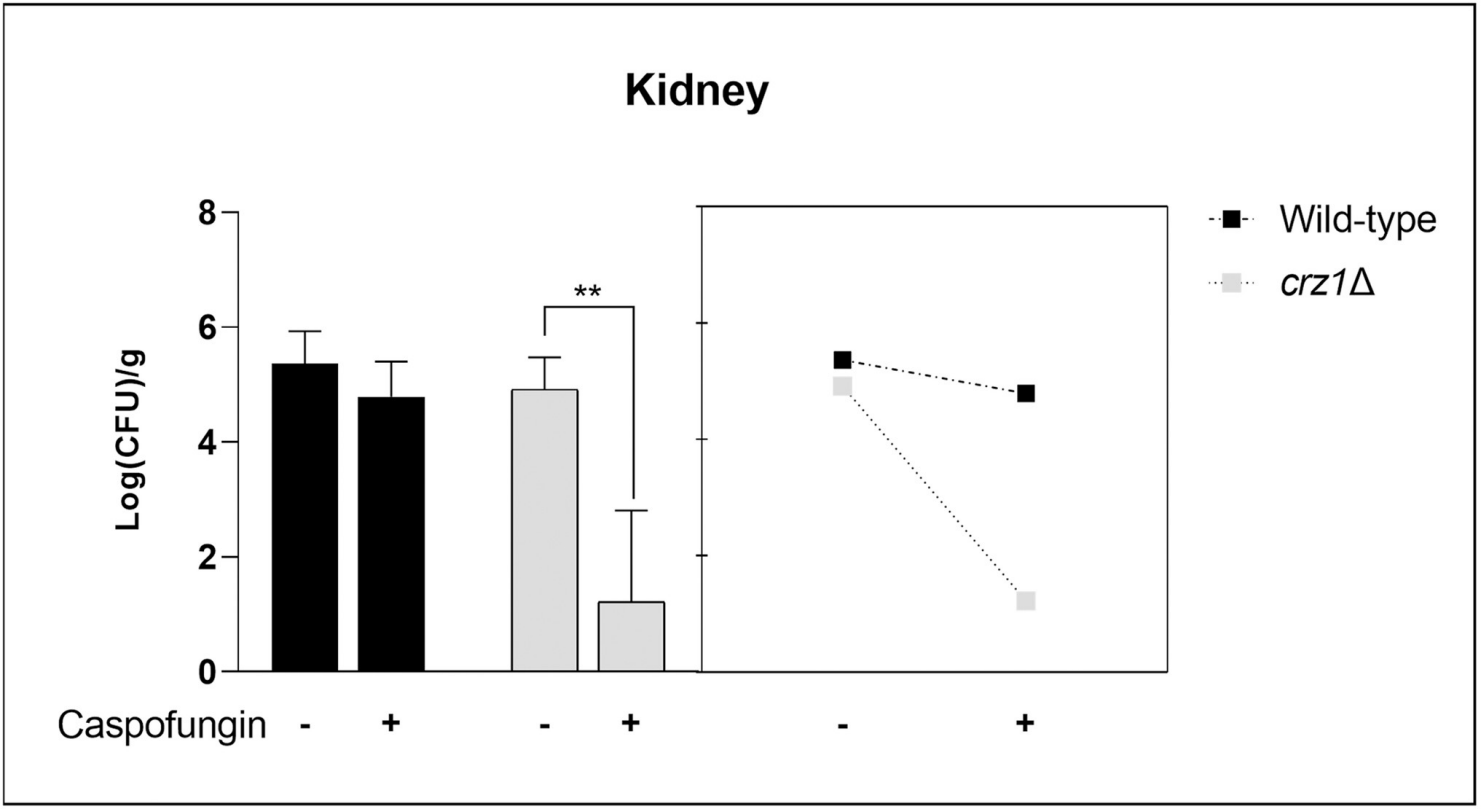
Fungal burden in kidneys from mice inoculated with 10^7^ yeast cells of caspofungin-resistant *C*. *glabrata* 1875 wild-type or *crz1*Δ strain and treated daily with (or without) caspofungin as indicated. Statistical analysis was carried out using the analysis of variance (two-way ANOVA) with post Tukey-Kramer test.

In a mouse model of disseminated candidiasis, 8 days after intravenously yeast inoculation, a high number of colony-forming units (CFUs) of the *C*. *glabrata* WT isolates were found in the kidney (10^5^ CFU/g) and the spleen (10^4^ CFU/g). These values were rather similar for *crz1*Δ mutants. As expected, the kidney fungal burden after infection with the caspofungin-resistant WT isolate was not affected by treatment with caspofungin. By contrast, caspofungin treatment led to a significantly lower fungal load (p < 0.005) in the group infected with the *crz1*Δ mutant (Fig 6). Regarding the number of CFUs in the spleen, no statistical differences were found between WT and mutant (p >0.005) (data not shown).

## Discussion

The spread of antifungal resistance and the limited number of available antifungal drugs amplify the need to identify new fungal targets for development of novel therapeutic alternatives. Here, we demonstrate that *CRZ1* disruption leads to echinocandin susceptibility in echinocandin clinical resistant *C*. *glabrata* isolates. This highly encouraging result could be supported by the well-known regulation of multiple biological processes governed by the CaM/CaL pathway and it is in agreement with previous studies showing the involvement of CaM/CaL and the downstream effector Crz1 in the antifungal response [26, 47, 48]. Indeed, glucan biosynthesis inhibitors exhibited fungicidal activity when combined with CaM/CaL inhibitors or by using calcineurin mutants of different *Candida* species [26, 49–51].

The ability of yeast cells to adapt to stress conditions and to activate cellular protection mechanisms represents an important survival strategy [52]. The CaM/CaL converges with other stress response pathways, such as the PKC and the PKA signaling pathways [53–55]. Here, *C*. *glabrata crz*1Δ mutants were subjected to heat stress and oxidative stress conditions. Our results suggest that although CaM/CaL is required for thermotolerance in *C*. *glabrata*, Crz1 is only involved in yeast growth at high temperatures, in concordance to what has been previously described [31, 47]. On the other hands, the antioxidant capacity of *C*. *glabrata*, mainly associated with the catalase Cta1, is higher than that of *S*. *cerevisiae* and *C*. *albicans*. Cta1 is controlled by the transcription factors Yap1, Msn2, and Msn4 and modulated by pathways other than the CaM /Cal [56]. Otherwise, our data show that Crz1 has a significant role in the oxidative stress tolerance in *C*. *glabrata* similarly to that reported for *Metarhizium acridum* [57]. This is an interesting result since it has been reported that the growth capacity in oxidative stress conditions was not affected by CaM/CaL inhibitors neither in calcineurin mutants [20, 55, 58] and therefore is a CaM/CaL-Crz1-independent phenomenon.

Regarding survival strategy, the fungal cell wall represents the first structure recognize by immune cell as and a key defense against all external attacks, including those mediated by the host immune system. Since the cell wall of fungi is not present in human cells, it is an attractive target for antifungal drug development [59]. Yeast cell walls are principally composed by glucans, chitin, and glycoproteins. Yeast remodels their cell wall over time in response to environmental changes, a process controlled mainly by the cell wall integrity (CWI) pathway [60]. The concept that the cell wall is dynamic and that structural rearrangements occur due to the upregulation of pressure-induced signaling pathways is not novel [59]. For example, in response to caspofungin, which induces a weakening of the cell wall, the yeast increases the production of chitin and/or mannan [61, 62]. For instance, we recently published the proteomic changes induced by the exposure to caspofungin in *C*. *glabrata*. We found that the proteins that were most abundant after caspofungin treatment were involved in DNA binding, i.e., CAGL0M06831g (Cg.Crz1) CAGL0J11440g (Ca.Srp1), CAGL0L10021g (Ca.Dbp5) and CAGL0C01683g (Ca.Isw1). Of interest are the proteins involved in antifungal responses, CAGL0M06831g (Cg.Crz1) (CaM/CaL-pathway), CAGL0J00539g (Cg.Slt2) (PKC-pathway) and CAGL0J11440g (Ca.Srp1) [55]. Moreover, in *C*. *albicans* caspofungin produced changes in expression of genes encoding cell wall maintenance proteins, including *FKS*, as well as *ECM21*, *ECM33*, *FEN12* and *PHR1* [63]. In this study, the *CRZ*1 disruption resulted in an apparent increase in chitin. Since Crz1 is involved in cell wall biogenesis and regulation of *FKS* genes, this may be a compensatory mechanism in response to cell wall destabilizing [26]. As described in *S*. *cerevisiae* [64].

*C*. *glabrata* normally produces blastoconidia measuring 2 to 8 microns in diameter. Interestingly, in c*rz1Δ* mutants, markedly enlarged "giant" (approximately 29 microns) blastoconidia were observed. Similar features related to cell size have been described in calcineurin mutants of *Mucor circinelloides* and for *Aspergillus flavus* conidia after calcineurin-Crz1 signalling pathway modulation [65, 66]. However, this giant cell production does not enhance *C*. *glabrata* virulence. Although we do not know the molecular mechanism for this phenomenon, we postulate that Crz1 disruption could lead to compensatory hyperactivation of the CWI and cAMP/PKA signaling pathways involved in cell size. Since, overexpression of PKA pathway members such as Pka1 caused an increase in cell size, moreover, the relation between Ca2+/CaM-PKA is well documented [67, 68]. In the dimorphic *M*. *circinelloides*, an emerging opportunistic pathogen, PKA activity is elevated during yeast growth in the presence of FK506, and in CaM/Cal mutants [65].

Biofilm formation is another important factor in the understanding of cellular adaptation.

Infections resulting from the formation of biofilms, if unsuccessfully managed, can have devastating consequences, progressing to IFI with a high risk of mortality [69, 70]. Then, the biofilm-forming capacity is one of the most important *Candida* virulence factors. Although CaM/CaL, HSP90 and mitogen-activated pathways have been associated with biofilm antifungal resistance acquisition, their role in biofilm development has been little studied [55, 70, 71]. In *C*. *glabrata*, adhesion step of biofilm formation is mediated by epithelial Epa- and Awp-adhesins (Awp1–13). The *EPA* family is composed of 23 genes, among which *EPA*1, *EPA*6, and *EPA*7 are the most relevant [59, 71]. In a previous study, we reported that fluphenazine/caspofungin combination reduced the ability of biofilm formation [20]. Here, we demonstrated by an *in vitro* model using polyurethane catheter pieces, that *CRZ1* disruption significantly affected biofilm development, confirming CaM/CaL-Crz1 pathway implication or CaM/Cal-Crz1 related pathways.

Deletion of *CRZ*1 reduces virulence in several human and plant pathogens such as *Aspergillus fumigatus* [66], *Cryptococcus neoformans* [72], *Magnaporthe oryzae* [73], and *Botrytis cinerea* [74]. In *M*. *acridum*, deletion of *CRZ1* leads to down-regulation of hydrophobins, proteins involved in cell surface hydrophobicity, adhesion and virulence [57]. Our data also showed that Crz1 had an important role in the pathogenicity of *C*. *glabrata* as was observed in the *Galleria mellonella* model.

Regarding the susceptibility pattern of *crz1* mutants, *in vivo* experiments confirm the *in vitro* susceptibility results. Indeed, caspofungin treatment reduces the larvae mortality and kidney fungal burden in the candidiasis disseminated models. However, as previously described caspofungin did not reduce the number of colonies of *crz1*Δ mutants in the spleen [75]. A significant change in the spleen fungal load was only observed with high dose of caspofungin and lower *C*. *glabrata* inoculum [76]. These observations could be due to the pharmacokinetic of caspofungin in the spleen which has already informed is superior in the liver and the kidneys, and in fact, is inferior to fluconazole and amphotericin B concentrations detected in the spleen [77, 78]. Moreover, in a murine model, infection with *cnb* mutants resulted in a greater reduction in fungal burden compared to *crz1* mutants. Interestingly, a decreased burden was observed in the kidney; unfortunately, *in vivo* antifungal activity was not evaluated [47].

Currently, drugs against CaM and Cal proteins exist and are used in humans as psychotropic and for immunosuppressive therapy, respectively. Nevertheless their potential use as antifungal therapy is not possible, due to the similarity of CaM/CaL targets between humans and fungi [24, 79]. Identification of novel selective drugs without side effects (e.g., immunosuppression) remains required. To achieve this objective, transcription factors are attractive as novel antifungal targets since they are evolutionarily divergent between fungi and humans and hence can be exploited as selective approach [80, 81]. Recently Malik et al, in an *in-silico* study described the potential antifungal properties of natural compounds against *Rhizoctonia solani* Crz1 [82], which confirms that the design of Crz1 inhibitors could be a successful therapeutic strategy for fighting life-threatening fungal diseases and increase of echinocandin resistance.

## Conclusions

In summary, the use of CRISPR-Cas9 allowed us to generate and evaluate phenotypically *crz1*Δ mutants from echinocandins-resistant *C*. *glabrata* isolates and to demonstrate their crucial role in *in vitro* and *in vivo* susceptibility to echinocandin, stress tolerance, biofilm formation, and virulence. In this sense, our results strongly suggest that inhibitors of Crz1 could have an important role in the development of novel therapeutic drugs to combat fungal infections, considering the increase of resistance phenomenon and the low number of antifungal drugs available.
